# HGF/c-Met axis drives cancer aggressiveness in the neo-adjuvant setting of ovarian cancer

**DOI:** 10.18632/oncotarget.2049

**Published:** 2014-06-01

**Authors:** Marisa Mariani, Mark McHugh, Marco Petrillo, Steven Sieber, Shiquan He, Mirko Andreoli, Zheyang Wu, Paul Fiedler, Giovanni Scambia, Shohreh Shahabi, Cristiano Ferlini

**Affiliations:** ^1^ Danbury Hospital Research Institute, Danbury, CT, USA; ^2^ Department of Gynecology, Catholic University of the Sacred Heart, Rome, Italy; ^3^ Department of Mathematical Sciences, Worcester Polytechnic Institute, Worcester, MA, USA; ^4^ Department of Oncology, Jean Paul IInd Research Foundation, Campobasso, Italy

**Keywords:** HGF, c-MET, Ovarian Cancer, Neo-Adjuvant Chemotherapy

## Abstract

Ovarian cancer is the most lethal gynecologic malignancy. Recently, NACT (Neo Adjuvant Chemotherapy) has been tested as alternative approach for the management of ovarian cancer patients. A biological predictor helpful in selecting patients for NACT would be desirable. This study was aimed at identifying actionable mechanisms of resistance to NACT.

Expression of a panel of microRNAs was screened in a discovery set of 85 patients. Analysis of the potential targets was conducted in the same RNAs by calculating significant correlations between microRNAs and genes. Quantitative fluorescent immunohistochemistry was employed in a validation set of 109 patients.

MiR-193a-5p was significantly overexpressed in the NACT setting. Analysis of its potential targets demonstrated that this microRNA is also significantly correlated with *HGF* and *MET* genes. Analysis of protein expression in samples taken before and after NACT demonstrated that both HGF and c-Met are increased after NACT. Patients who relapse shortly after NACT exhibited the highest relative basal expression of both HGF and c-Met, while the opposite phenomenon was observed in the best responders.

Mir-193a-5p, HGF and c-Met expression may help select eligible patients for this modality of treatment. Moreover, inhibitors of this pathway may improve the efficacy of NACT.

## INTRODUCTION

Ovarian cancer is the leading cause of death for gynecologic malignancies. In the United States, it is estimated that approximately 22,200 new cases will be diagnosed in 2013 and 15,550 deaths will be reported due to this cause. The high rate of mortality relates to presentation at advanced stage in roughly 85% of patients. The standard protocol of treatment for ovarian cancer includes maximal cytoreductive surgery (primary debulking surgery, PDS) followed by platinum/taxane chemotherapy (PDS-CT). Although the majority of the patients will exhibit a response to PDS-CT, relapse with resistance to additional treatments is common. Fatal progression of ovarian cancer is sadly the norm, with a five year survival rate in this disease of just 20-30% and a ten year survival rate below 10% [1].

In the last decade, a therapeutic treatment alternative to PDS-CT has been developed. In this scenario, neo-adjuvant chemotherapy (NACT) is delivered before debulking surgery which is then followed by an additional round of chemotherapy. This alternative therapeutic option, initially reserved only for unresectable patients, is now increasingly utilized. A recent clinical trial (EORTC55971) has demonstrated that, in terms of overall survival, NACT is comparable to PDS-CT, while offering a lower complication rate and faster recovery after debulking surgery [2]. Other clinical studies are in line with the results of the EORTC55971 [3], and support the notion that NACT is associated with fewer post-surgical complications. Therefore, it appears possible that NACT will become an alternative treatment offered to a broader number of patients in the near future [4]. However, other studies are contradictory on this topic and still support a more conservative approach to NACT [5]. This hesitation is mostly driven by the fact that exposure to chemotherapy may reduce the ability to visualize cancer during surgical tumor debulking while at the same time selecting for survival of the most aggressive, drug-resistant, cancer cells [6].

While the role of NACT is evolving in the clinical arena, this treatment modality provides a unique opportunity to investigate the biology of ovarian cancer response to chemotherapy and the molecular mechanisms which may be involved in the emergence of drug-resistance. To exploit this opportunity, we compared microRNA and gene expression of potential actionable targets in patients treated with NACT as compared to those treated with PDS-CT. Thereafter, we validated the results at the protein level using fluorescent quantitative immunohistochemistry. Results indicate that hepatocyte growth factor (HGF) and its receptor c-Met are significantly increased post-NACT patients, thus representing potential molecular targets for combination chemotherapy in this population.

## RESULTS

### Discovery set analysis of gene and microRNA expression

**Fig. 1:**
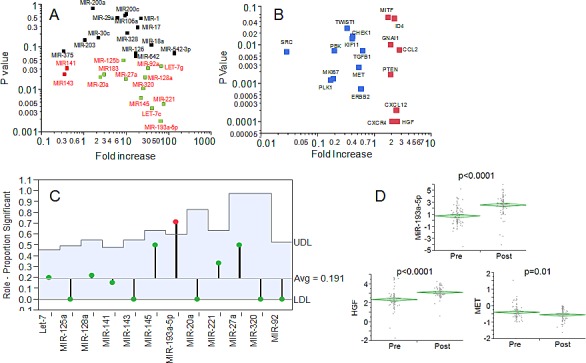
A: Dot plot showing the results of microRNA expression in the discovery set. In x- and y-axis the fold increase (NACT/PDS-CT) is reported along with the statistical significance calculated with Wilcoxon test. In black, red and green are reported the microRNAs not significantly modulated, significantly decreased in NACT and significantly increased in NACT, respectively. B: Dot plot showing the results of gene expression analysis in the discovery set. In x- and y-axis the fold increase (NACT/PDS-CT) is reported along with the statistical significance calculated with Wilcoxon test. Only the genes (red and blue are upregulated and downregulated in NACT, respectively) significantly modulated are shown. C: ANOM analysis of the target significantly modulated in NACT. Y-axis indicates the perecentages of significant target for each microRNA. The blue area indicates the upper/lower decision level for each micro-RNA. Only miR-193a-5p exhibited a significant proportion of targets modulated in the NACT setting. D: Diamond chart showing the expression of miR-193a-5p, HGF and MET in the validation set of 109 NACT patients. The top and bottom of each diamond represent the confidence interval for each group mean. The mean line across the middle of each diamond represents the group mean. A statistically significant increase was noticed for both miR193a-5p and HGF. A significant decrease was also reported for MET (Wilcoxon assay).

A clinical cohort of 85 ovarian cancer patients, whose clinical features are summarized in Table 1, was enrolled in a retrospective analysis as discovery set. Sixty-three patients were treated with traditional PDS-CT while twenty-two underwent NACT. For both categories of patients, a paraffin block of tumor from the first surgery was analyzed. While the PDS-CT patients were previously untreated, the NACT patients received from three to six cycles of standard chemotherapy (platinum/taxane) before debulking surgery. We hypothesized that under the pressure of chemotherapy, the most resistant tumor cell clones would show preferential survival and be over-represented in the sample. In order to identify the biological circuits underlying resistance (and subsequent disease progression) in the face of chemotherapy, we performed microRNA analysis. MicroRNAs can regulate hundreds of genes and provide clues regarding a multitude of potential molecular pathways involved in this phenomenon. We chose twenty-eight microRNAs whose expression has been recently related to ovarian cancer cell drug resistance [7]. All the microRNAs were analyzed with a nanofluidic genetic analyzer which made possible for the low volume of reaction (10 nanoliters) to perform microRNA and gene expression on the same RNA sample. The results of all the patients were grouped according to treatment (either PDS-CT or NACT), and statistically significant differences (p-value <0.05) were computed with the use of Wilcoxon test. As shown in Fig. 1A, two microRNAs (miR-141 and miR-143) were downregulated and twelve (miR-20a, miR-183, miR-125b, miR-27a, mir-92s, let-7g, miR-128, miR-320, miR-145, miR-221, let7c and miR-193a-5p) were upregulated in the NACT setting. MiR-193a-5p exhibited the most significant upregulation.

**Table I:** Clinical Features of the analyzed setting of ovarian cancer patients

Characteristics	Number (%) Discovery Set	Number (%) Validation Set

Cases	85	109
Age, yrs Median	64	61
FIGO Stage I-II III IV	15 (17.6) 65 (76.1) 5 (5.8)	0 (0) 86 (78.9) 23 (21.1)
Histotype Papillary-serous Mucinous Endometrioid Clear Cell Undifferentiated	72 (83.6) 3 (4.0) 7 (9.3) 2 (2.7) 1 (1.3)	97 (88.9) 0 (0) 4 (3.7) 4 (3.7) 4 (3.7)
Ca 125 Median (range)	309 U/mL (13-34000)	972 U/mL (30.5->10000)
Status Dead Alive Median follow up (Alive)	31 (36.5) 54 (63.5) 58 months	65 (59.6) 44 (40.4) 42 months
Response Refractory Resistant Sensitive		15 (14) 54 (51) 37 (35)

All the significant 14 microRNAs were analyzed for potential actionable targets using the microRNA.org search engine. Each gene was prioritized for scoring and then screened in the Genecard and Pubmed database for the presence of a correspondent drug inhibitor. Eighty target genes were chosen according to such criteria. The genes *DROSHA* and *DICER* were included in the analysis for the average higher levels of expression of microRNAs observed in the NACT setting. Results for all the genes are reported in Supplementary Table 1 while the results of eighteen targets, significantly modulated in the NACT setting, are shown in Fig. 1B. Among all the genes significantly regulated in the NACT population, ten exhibited an inverse correlation (ρ<0:*MKI67, CHEK1, SRC, MET, PBK, PLK1, ERBB2, TWIST1, KIF11, TGFB1*) and eight a direct correlation (ρ>0: *MITF, ID4, CXCR4, CXCL12, GNAI1, CCL2, PTEN, HGF*). Analysis of the mean proportions [8] revealed that the targets of miR-193a-5p were significantly more represented in the NACT group than in PDS-CT, thus suggesting that this microRNA is one of the key drivers modulating gene expression in NACT patients (Fig. 1C).

### Gene and microRNA expression validation in 109 patients pre/post NACT treatment and in the TCGA dataset

NACT and PDS-CT patients tend to have not overlapping clinical features since NACT is preferentially reserved for patients featuring high tumor dissemination unresectable at first diagnosis [9]. In order to validate the results obtained in the discovery set we enrolled an additional clinical cohort (validation set) of 109 patients. In order to overcome a potential confounder effect driven by clinical differences between NACT and PDS-CT women, we compared the expression of the three factors (miR-193a-5p, *HGF* and *MET*) in a clinical cohort all treated with NACT (Tab. 1), with samples collected at first diagnosis (pre-NACT) and at the interval debulking surgery (post-NACT). MiR-193a-5p was again remarkably increased after NACT (Fig. 1D). Similarly, also *HGF* and *MET* genes were significantly upregulated and downregulated after NACT, respectively (Fig. 1D). These results confirmed the validity of the observations we made in the discovery set for these three variables.

**Fig. 2:**
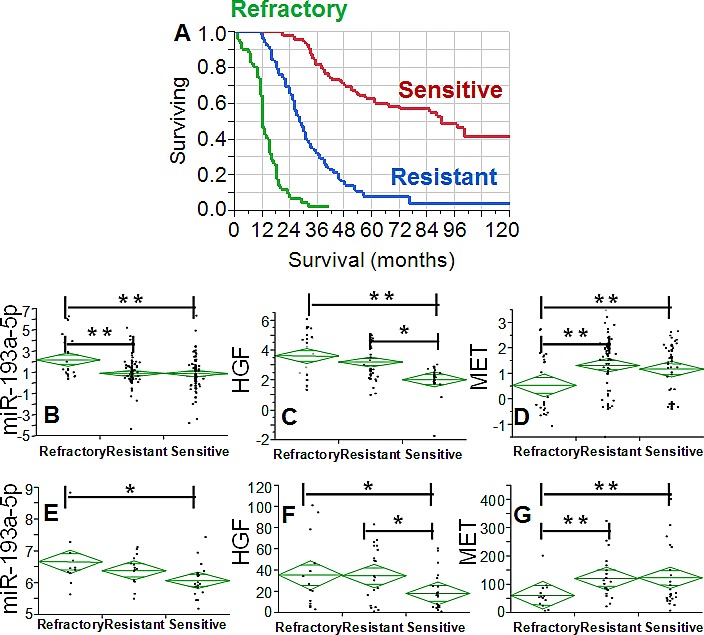
A: Kaplan-Meier curves of the survival of the validation setting for patients grouped according to PFI. Green, blue, and red line is the survival curve for patients belonging to the refractory (PFI 0-3 months), resistant (PFI 3-12 months) and sensitive setting (PFI >12 months). Difference among the three groups is highly significant (p<0.001, Wilcoxon test). B-D: Diamond chart showing the expression of miR-193a-5p (B), *HGF* (C) and *MET* (D) according to response to NACT. The top and bottom of each diamond represent the confidence interval for each group mean. The mean line across the middle of each diamond represents the group mean. E-G: Diamond chart reporting the expression of miR-193a-5p (E), HGF (probe 210997_at, F) and MET (probe 211599_x_at, G) in the TCGA dataset. Analysis was restricted to stage IV patients. The top and bottom of each diamond represent the confidence interval for each group mean. The mean line across the middle of each diamond represents the group mean. In B-G double and single asterisks indicate a significant difference at a p value <0.001 or <0.05, respectively (Wilcoxon test).

In order to connect these findings with response to NACT, the expression of miR-193a-5p, *HGF* and *MET* was analyzed after grouping patients according to platinum free interval (PFI). We categorized patients into three groups according to PFI as refractory (PFI<3 months), resistant (PFI 3-12 months) and sensitive (PFI>12 months). PFI represents the time between last platinum therapy and disease relapse and is a known correlate of overall survival in ovarian cancer [10]. Our clinical setting was not different and outcome was driven by PFI status (Fig. 2A). The expression of miR-193a-5p was increased in the refractory group (Fig. 2B) and accompanied by a concomitant increase of *HGF* (Fig. 2C) and a decrease of *MET* (Fig. 2D).

To our knowledge this is the first study in which miR-193a-5p, *HGF* and *MET* are analyzed in NACT patients, thus making difficult external crossvalidation of our findings. In order to support our results, we analyzed the expression of the three factors in the TCGA dataset [11]. As a proxy of our clinical subset, we restricted the analysis to patients with stage IV (n=58), which is a clinical setting with a disease so extended which would be treated with NACT in our Institution. For this reason, we assumed that this clinical cohort is very similar to the pre-NACT patients since the TCGA samples were collected before chemotherapy. Analysis was performed after grouping patients according to PFI (refractory, resistant and sensitive) as described above. MiR-193a-5p levels were again higher in the refractory group as compared with those noticed in the other groups (Fig. 2E). Similarly, *HGF* expression was even in this clinical setting significantly increased in the refractory group (Fig. 2F), while *MET* levels were significantly decreased only in the refractory setting (Fig. 2G), thus mirroring the trend we noticed in our validation set.

### *In vitro* validation of *HGF* and *MET* as targets of miR-193a-5p

**Fig. 3:**
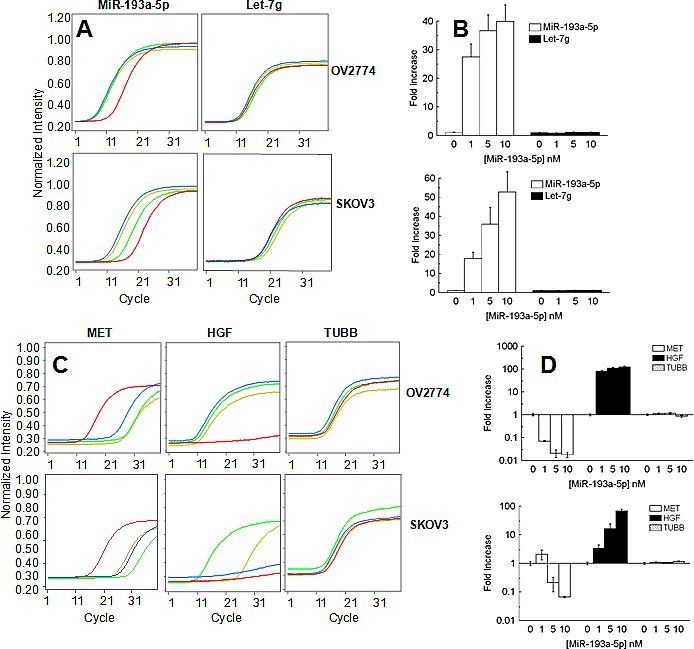
A: Representative qPCR analysis of the expression of miR-193a-5p (left column) and let-7g (right column) in OV2774 (top) and SKOV3 (bottom). The red lines marks the cells treated with the transfecting medium, while green yellow and blue lines are for 1, 5 and 10 nM of transfected miR-193a-5p after 48 hours of culture, respectively. B: Bar chart reporting the results of two independent experiments performed in triplicate samples. Expression was normalized for the negative control (only transfection medium=1). Bar and error bars represents mean and SD, respectively. C: Representative qPCR analysis of the expression of MET (left column), HGF (middle column) and TUBB (right column) in OV2774 (top) and SKOV3 (bottom). The red lines marks the cells treated with the transfecting medium, while green yellow and blue lines are for 1, 5 and 10 nM of transfected miR-193a-5p after 48 hours of culture, respectively. D: Bar chart reporting the results of two independent experiments performed in triplicate samples. Expression was normalized for the negative control (only transfection medium=1). Bar and error bars represents mean and SD, respectively.

In two ovarian cancer cell lines (SKOV3 and OV2774) we transfected a biotin tagged miR-193a-5p at three concentrations (1, 5 and 10 nM, Fig. 3A-B). A sample with only the transfecting medium was kept as negative control while the expression of let-7g was kept as a reference. After 48 hours, we analyzed endogenous *HGF* and *MET* gene expression to assess the changes induced by overexpression of the microRNA. In keeping with the observations in patients reported above, in both cell lines miR-193a-5p augmented the levels of *HGF* and concomitantly repressed *MET*, while the expression of a non-target gene like TUBB remained unchanged (Fig. 3C). In order to test the physical association between miR-193a-5p and *HGF/MET* genes, the synthetic microRNA was pulled down using streptavidin beads. A library of cDNA was prepared from OV2774 transfected with 10nM of the microRNA and from the negative control. The presence of *HGF* and *MET* products was ascertained with PCR, while no presence of TUBB gene was detected (Fig. 3D and Supplementary Fig. 1). Altogether these results support that both *HGF* and *MET* are modulated by miR-193a-5p through a direct interaction.

### Protein analysis in the validation set of 109 patients pre/post NACT

**Fig. 4:**
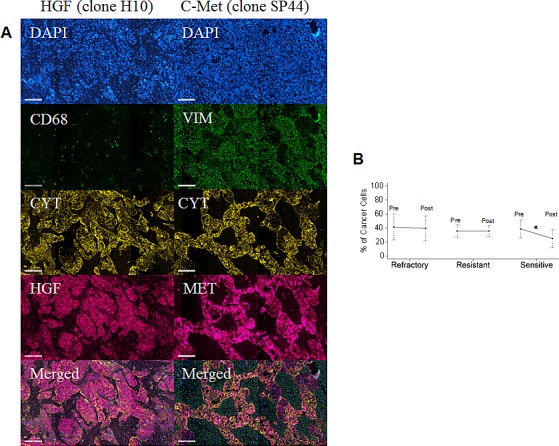
A Representative fluorescent immunohistochemistry for HGF (left column) and c-Met. From top to bottom; DAPI channel, CD68 (HGF) and vimentin (c-Met) channel, pan-cytokeratin, HGF and c-Met, merged image. As expected, cytokeratin and vimentin/CD68 have a non-overlapping pattern of staining, while HGF and c-Met are expressed in both cancer and macrophage or stromal compartments. B: Chart summarizing the number of cancer cells in specimens used for proteomic analysis in the validation set. Data point and bar represent the average and the 5^th^-95^th^ percentile range. Patients were grouped according to PFI and the number of cancer cells in each specimen quantified using the cytokeratin mask. There was no difference in the number of cancer cells in the refractory and resistant group. There was a significant decrease of cancer cells (p<0.05, Wilcoxon test) in the patients with sensitive disease.

**Fig. 5:**
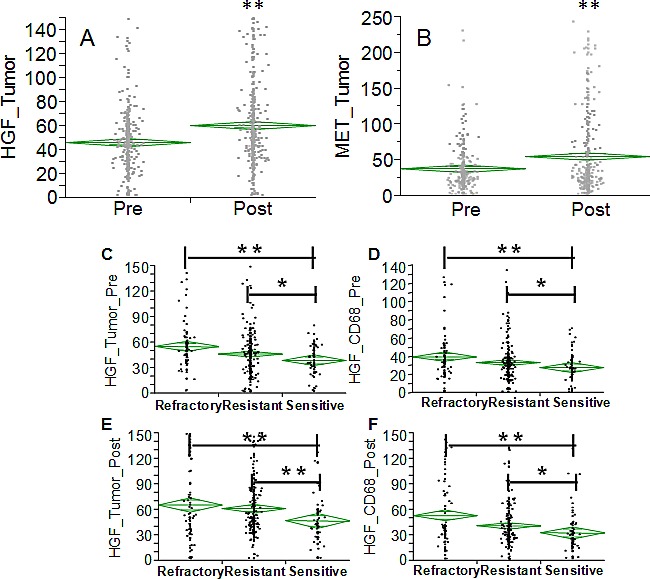
A-B: Diamond chart reporting the expression of HGF (A) and c-Met (B) in specimens collected before and after NACT (validation set). The top and bottom of each diamond represent the confidence interval for each group mean. The mean line across the middle of each diamond represents the group mean. Double asterisks indicate a strong statistical significance of the difference (p<0.001, Wilcoxon test). C-E Diamond chart reporting the expression of HGF in specimens (validation set) collected before (C-D) and after (E-F) NACT in cancer cells (C&E) and macrophage CD68+ (D&F). The top and bottom of each diamond represent the confidence interval for each group mean. The mean line across the middle of each diamond represents the group mean. Double asterisks indicate a strong statistical significance of the difference (p<0.001, Wilcoxon test). Single asterisk indicates significant difference at a p value <0.05.

**Fig. 6:**
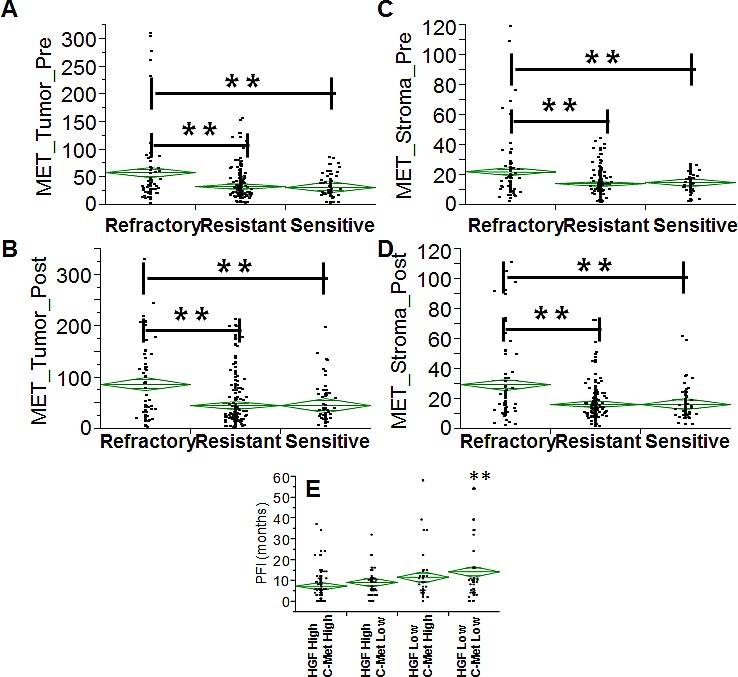
A-D Diamond chart depicting the expression of c-Met in specimens (validation set) collected before (A-C) and after (B-D) NACT in cancer cells (A-C) and stromal cells which are vimentin+ (B-D). The top and bottom of each diamond represent the confidence interval for each group mean. The mean line across the middle of each diamond represents the group mean. Double asterisks indicate a strong statistical significance of the difference (p<0.001, Wilcoxon test). E: diamond chart depicting the PFI (y-axis) across four groups (double positive, double negative for HGF/c-Met, single positive for HGF or c-Met). Double asterisks indicate a strong statistical significance of the difference (p<0.001, Wilcoxon test, double positive vs. double negative).

In order to extend the analysis to the protein dimension, TMAs were prepared from the same tumor specimens reported above. Analysis was performed in triplicate cores to probe clonal heterogeneity inside the specimen in both pre and post-NACT specimens. Analysis was performed in independent replicate slides using quantitative fluorescent immunohistochemistry in multiplexing using DAPI as a nuclear stain (blue channel), CD68 as a marker of macrophages in the HGF analysis or vimentin as a marker of stromal cells in the c-Met analysis, pan-cytokeratin as a marker of epithelial tumor cells and the two protein antigens of interest (red channel). A representative image is depicted in Fig. 4A. Analysis was quantified using the AQUA^®^ software. The system utilizes an unsupervised method to calculate expression of the antigens using a predefined set of algorithms capable of scoring the expression in cellular masks of interest. In our study, we selected two alternative mask pairs: tumor (cytokeratin) and macrophage (CD68+) for the HGF analysis and tumor (cytokeratin) and stroma (vimentin+) for the c-Met analysis. As preliminary approach the number of cancer cells (cytokeratin positive) was scored in each specimen coming from pre- and post-NACT. Average number of cancer cells for specimen did not change in the refractory and resistant groups post NACT, while in women with chemo-sensitive disease there was a decreased presence of tumor cells after NACT (Fig. 4B). For that concerning HGF, staining was present in both tumor and macrophages and, similarly, c-Met expression in both tumor and stromal cells. Correlating gene and protein expression according to response to NACT, HGF was significantly increased in patients after NACT (Fig. 5A), while in contrast to the gene expression data, c-Met was also significantly increased after NACT (Fig. 5B). In samples taken before NACT, HGF expression was significantly lower in sensitive patients as compared with women with refractory or resistant disease. The trend was similar in both cancer (Fig. 5C) and CD68+ cells (Fig. 5D). Post treatment, again the sensitive group showed a significantly lower expression of HGF as compared with the others (Fig. 5 E&F). On the other hand, c-Met was significantly higher in the refractory group pre-NACT (Fig. 6 A&C). This difference was accentuated post treatment in both tumor and stromal cells (Fig. 6 B&D). Altogether, these findings suggest that high relative expression of both HGF and c-Met are associated with refractory disease, whereas low expression of both HGF and c-Met are related to a favorable response to NACT. To validate these findings, we re-classified patients into four subgroups for analysis of PFI as continuous variable: High HGF/High c-Met, High HGF/Low c-Met, High c-Met/Low HGF and Low HGF/Low c-Met. PFI (Fig. 6E) was significantly shorter in the group with High HGF/High c-Met (7.1 months as compared with the 13.4 months for patients with Low HGF/Low c-Met). These results suggest that high expression of miR-193a-5p is associated with high levels of both HGF and c-Met proteins.

## DISCUSSION

Ovarian cancer is the most lethal gynecologic malignancy. Since the advent of standard first line chemotherapy, no significant improvement in outcome has been obtained [12] and the most compelling clinical problem is represented by drug-resistance and progression upon chemotherapy. This problem is particularly urgent in NACT which is delivered to reduce tumor burden before debulking surgery. In fact, NACT will be useless to reduce tumor mass if the patient will be refractory to chemotherapy and progress during the treatment.

In this study, we exploited NACT as a tool to study the mechanism underlying drug resistance due to the opportunity to sample and analyze surviving tumor cell clones post-chemotherapy in a cohort of patients. Moreover, we focused our investigation on actionable molecular targets in the hopes of enhancing the translational value of our study and identify a rationale useful to complement NACT with a targeted agent possibly useful to increase the response rate in the refractory group. We employed an integrated approach including gene/microRNA expression coupled with quantitative fluorescent immunohistochemistry. Analysis started with a panel of prognostic microRNAs [7]. With the exception of miR-141 and miR-143 we identified a broad increase of microRNA levels in NACT patients and a particularly significant increase of miR-193a-5p. This microRNA is a central regulator of platinum response in squamous cell carcinoma and it is overexpressed as a consequence of the DNA damage induced by chemotherapy [13]. ANOM analysis indicated miR-193a-5p as a central regulator of the changes noticed at the gene levels in ovarian cancer patients after NACT. Among the factors significantly correlated with miR-193a-5p, we noted both *HGF* and *MET* genes. HGF is a pleiotropic factor involved in the enhancement of metastatic potential of cancer cells [14] and *MET* encodes for its cellular receptor c-Met. Their expression seems enhanced in aggressive ovarian cancer [15], although some conflicting data has been published [16-18]. In our study, we demonstrate for the first time that the expression of both HGF and c-Met are modulated under the pressure of chemotherapy in NACT patients. Noteworthy, the HGF/c-Met pathway seems significantly altered in terms of expression levels in patients who subsequently are refractory to NACT, with a PFI ≤ 3 months. Conversely, patients with low relative expression of HGF and c-Met tended to have longer PFI and a better outcome. These findings suggest that this pathway could be exploited as an additional tool to complement clinical criteria of eligibility for NACT. Indeed, if a patient exhibits a refractory disease, it is very unlikely that she will benefit from NACT treatment, which will be unable to reduce the tumor mass and makes the surgical debulking easier than pre-NACT. Furthermore, the HGF/c-Met axis is actionable with a series of targeted strategies [15]. Due to the direct involvement of HGF/c-Met in ovarian cancer aggressiveness, we believe that our data support the rationale for clinical trials of inhibitors of the HGF/c-Met axis in the context of the NACT setting. These inhibitors would be of particular value in patients for whom NACT is the only viable treatment option due to extensiveness of disease at presentation such as stage IV.

The strength of the presented results is coming from the integration of genomic and proteomic approach. If gene and microRNA expression can be performed in a relatively high-throughput way, there is always the problem of connecting the obtained results with the expression in individual subset of cells (cancer/stromal) which are represented in different proportions in a specimen. This problem is solved by proteomic analysis with quantitative fluorescent immunohistochemistry. Using such approach antigens of interest are quantified in the different subsets of cells regardless of the absolute amount of cancer/stromal cells in a sample. It is also important to note that proteomic analysis is mandatory in order to drive clinical decisions around the use of specific targeted agents. In fact, we cannot predict from the trends in gene expression which will be the impact at the protein level, since a high correlation between gene and protein expression is present in the majority but not all the genes in human and mouse cells [19]. In order to be actionable, the relevant element is not the gene but the protein, which ultimately will be targeted with a specific drug. In our analysis, we reported that HGF belongs to the category of factors in which the trend between gene and protein expression is the same. On the opposite, MET does not follow the same rule. If the expression at the gene level is decreased, in the same specimens the expression of the protein is increased. How a similar trend can be explained? At variance with other solid malignancies, in ovarian cancer c-Met overexpression is not driven by genetic amplification [20]. For this reason, c-Met inhibitors with multikinase activity may exhibit less activity in ovarian cancer than c-Met specific drugs [21]. In this context it is possible that c-Met increased expression is a mechanism of adaptation to hypoxia and poor angiogenesis as recently demonstrated with the use of anti-VEGF therapy [22]. The same mechanism has been proposed in metastatic breast cancer where HGF/c-Met axis is exploited to counteract the functional consequences of hypoxia [23]. These findings together support the potential clinical utility in the refractory NACT setting (exhibiting high HGF (protein/gene) and low *MET* gene high c-Met protein) of the inhibition of both c-Met and HGF. It has been demonstrated that a block of translation can produce an apparent upregulation of the most transcribed genes, thus demonstrating a feedback loop between translation and mRNA degradation [24-26]. On the opposite, enhancement of translation can be accompanied by decrease of mRNA stability and consequent reduced mRNA levels [24]. However, in this context a caveat in our analysis is that we did not have a separate validation set for the noticed changes at the protein level. In this sense, our results need to be confirmed in future prospective clinical trials.

In summary, this translational study demonstrates that high protein expression levels of HGF/c-Met are present in patients who will be refractory to NACT. This finding potentially creates an opportunity to improve diagnostic and therapeutic strategies for a clinical category of ovarian cancer patients who minimally benefit from chemotherapy and debulking surgery.

## MATERIAL AND METHODS

### RNA extraction from FFPE

Two clinical cohorts were analyzed in this retrospective study. A discovery set (n=85) was composed by 63 and 22 patients treated with PDS-CT and NACT, respectively. A validation set (n=109) was composed by patients all treated with NACT. Clinical features of the two cohorts are summarized in Tab. 1. After approval of the Danbury Hospital Internal Review Board and collection of the relevant clinical information, de-identified FFPE samples were obtained from ovarian cancer cases that had been preserved between 2000 and 2008. FFPE samples were cut to 10 μm thickness and two tissue slices were put into a 1.5 ml tube. To each tube, one milliliter of xylene was added for deparaffinization followed by mixing twice with a high speed vortex for 3 min at room temperature. Total RNA was then automatically extracted with the QIAcube using the miRNeasy FFPE kit (Qiagen, Valencia, CA) following manufacturer’s protocol. The RNA from A2780 and OVCAR-3 cells was automatically extracted with the QIAcube using the miRNeasy kit (Qiagen, Valencia, CA) following manufacturer’s protocol. RNA quantity and the quality were assessed by Agilent 2100 Bioanalyzer (Agilent Technologies, Santa Clara, CA). RNA from the cell lines was used as a reference.

### Gene expression analysis

Total RNA was reverse transcribed using High Capacity cDNA Reverse Transcription Kit (Applied Biosystem, Foster City, CA). The 20 μl reverse transcription reaction contained 10 μl of total RNA, 0.8 μl of 100 nM dNTP, 1 μl of RNase inhibitor 20 U/μl, 1 μl of reverse transcriptase (50 U/μl), 2 μl of 10X RT random primers, 2 μl of 10X RT buffer and 3.2 μl of ultrapure H_2_O. The reaction mixture was mixed with RNA and incubated as follows: 25°C for 10 min, 37°C for 120 min and then 85°C for 5 min. For pre-amplification of cDNA, TaqMan assays were pooled at a final concentration of 0.2X for each assay. The pre-amplification PCR was performed at one cycle 95°C for 10 min, 14 cycles at 95°C for 15 sec and then 60°C for 4 min. After pre-amplification PCR, the product was diluted 1:5 with DNA Suspension Buffer and stored at -20°C until needed. Preparation of the chip was performed following the manufacturer’s protocol on a BioMark system (Fluidigm, South San Francisco, CA). Briefly, an IFC controller was used to prime the fluidics array chip with control line fluid (~15 min). Samples and assays were loaded into the 48.48 dynamic array chip (from Fluidigm Corporation) by inserting the chip into the IFC controller. The chip was then loaded onto the BioMark Instrument and the reaction was performed at one cycle 50°C for 120s, one cycle 95°C for 10 min, 40 cycles at 95°C for 15 sec and 60°C for 4 min. Data analysis was performed using the real-time PCR Analysis Software of the Biomark platform (Fluidigm Corporation, CA, USA) using the delta-delta ct method as previously reported [27, 28]. The TCGA dataset was downloaded from the TCGA website (http://cancergenome.nih.gov). Level 2 gene expression data derived from Affymetrix U133A platform were used to infer the expression of mir-193a-5p, *HGF* and *MET*. Analysis was restricted to stage IV patients (n=58) and clinical information was downloaded from the TCGA website.

### MicroRNA expression analysis

Total RNA was reverse transcribed using the TaqMan MicroRNA Reverse Transcription Kit (Applied Biosystem, Foster City, CA) with the Megaplex RT Primers, Human Pool A v2.1 (Applied Biosystem, Foster City, CA). The reaction mixture was mixed with RNA and incubated as follows: one cycle 16°C for 2 min, 40 cycles at 42°C for 1 min and 50°C for 1 sec, and then 85°C for 5 min. For pre-amplification of cDNA, the Megaplex PreAmp Primers (Applied Biosystem, Foster City, CA) were used. Pre-PCR amplification reaction was done at 5 μl containing 2.5 μl TaqMan PreAmp Master Mix (2X), 0.5 μl of 10X Megaplex PreAmp Primers and 2 μl of cDNA. The pre-amplification PCR was performed at one cycle 95°C for 10 min, one cycle 55°C for 2 min, one cycle 72°C for 2 min, 18 cycles at 95°C for 15 sec and 60°C for 4 min, and then one cycle 99.9°C for 10 min. After pre-amplification PCR, the product was diluted 1:10 by adding 45 μl of DNA Suspension Buffer and stored at −20°C until needed.

Briefly, a 5 μl sample mixture was prepared for each sample containing 1 × TaqMan Universal Master Mix, 1X GE Sample Loading Reagent (Fluidigm PN 85000746) and each of diluted pre-amplified cDNA. Five μl of Assay mix was prepared with 1X each of TaqMan miRNA assay and 1X Assay Loading Reagent (Fluidigm PN 85000736). An IFC controller was used to prime the fluidics array (chip) with control line fluid and then with samples and assay mix in the appropriate inlets. After loading, the chip was placed in the BioMark Instrument for PCR at 95°C for 10 min, followed by 40 cycles at 95°C for 15 sec and 60°C for 1 min. Data analysis was performed using the real-time PCR Analysis Software of the Biomark platform (Fluidigm Corporation, CA, USA) using the delta-delta ct method as previously reported [27, 29]. Three small RNAs were used as loading control (RNU44, RNU46, MAMMU6) while the cell line A2780 was used as a reference.

### Transfection and pull down assay using biotinylated microRNA Mir-193a-5p

Biotinylated Mir-193a-5p was obtained from Eurofins MWG Operon as a miRNA duplex in which the sense filament, at the 3’ end, was labeled with a biotin. The tag used, called Biotin-TEG, was linked to the miRNA through a 15-atom triethylene glycol spacer. Both sense and antisense strands carried a 2-nt 3’ overhang, to increase the target sensitivity of siRNA [30]. The sequence of the duplex Mir-193a-5p is: 5’- UGGGUCUUUGCGGGCGAGAUGAUU-3’ and 3’-UCAUCUCGCCCGCAAAGACCUAGA-5’.

OV2774 and SKOV3 cells were seeded in 6 well dishes, 2x10^6^ cell/well, for 48h without reaching the full confluency. These cellular models were chosen since preliminary data demonstrated low expression of miR-193a-5p, high expression of MET and low expression of HGF in the panel of cell lines available in our laboratory. HiPerFect transfection reagent (Qiagen, Valencia, CA) was used to transfect the cells with the biotin-tagged miRNA at final concentration of 1, 5 and 10nM. A transfection with only HiPerFect reagent represented the negative control. For each cell line RNA extraction and microRNA/gene expression was performed as described above. Using dynabeads MyOne Streptavidin C1(Life Technologies, Carlsbad, CA) as described by Orom & Lund [31] a pull down assay of the Mir-193a-5p was performed. The total RNA extraction from the dynabeads was executed using rapid homogenization spin-column Qiasheredder and RNeasy mini KIT (Qiagen, Valencia, CA) following the manufacturers protocol. The RNA was utilized to prepare a library of cDNA pulled down as described by Kurimoto and colleagues [32]. To prove the physical interaction between Mir-193a-5p and HGF and MET messengers, a PCR reaction was performed using TaqMan Universal PCR Master Mix (Applied Biosystem, Foster City, CA) with *HGF*, *MET* and *TUBB* TaqMan Gene Expression Assays (Applied Biosystem, Foster City, CA). The reaction mixture was mixed with cDNA and incubated as follows: one cycle 50°C for 2 min, one cycle 95°C for 10 min and then 40 cycles at 95°C for 15 sec and 60°C for 1 min. The profile of the PCR products was evaluated by using the Agilent 2100 Bioanalyzer (Agilent Technologies, Santa Clara, CA).

### Quantitative fluorescent immunohistochemistry

Tissue specimens were prepared in a TMA format: representative tumor areas were obtained from FFPE specimens of the primary tumor, and up to three representative replicate 2-mm cores from multiple tumor blocks were taken after review and marking of the hematoxylin and eosin stained slides by board-certified pathologists (SS and PF). In total, 660 cores were taken and distributed over 11 slides from 109 patients, all of whom having pre and post-NACT paired samples. Staining of HGF was obtained with clone H-10 anti-HGF antibody (Santa Cruz Biotech, Santa Cruz, CA). Validation of the antibody was performed in OVCAR-3 cells (no expressing HGF) and its patupilone resistant counterpart with high expression of HGF [33]. Additional validation was performed in tissues using fetal liver as a positive control. The clone SP44 was chosen to stain c-MET for the fact that has been selected to enroll patient eligible for treatment with Rilotumumab in a phase III clinical trial (http://clinicaltrials.gov/show/NCT01697072). A detailed protocol for staining and analysis is provided in supplementary methods.

### Statistical and Bioinformatic Analysis

The significance of increased/decreased expression of a microRNA or a gene (NACT vs. PDS-CT groups) was calculated using Wilcoxon test and a p value <0.05 as a threshold of significance. The fold increase was established dividing the value noticed in NACT over that of PDS-CT, thus meaning that positive and negative values are related to increase and decrease, respectively. The gene list of putative targets of microRNAs was prepared using diverse online softwares including in the microRNA.org website, such as Targetscan (www.targetscan.org) and PicTar (http://pictar.mdc-berlin.de/). The list was then processed using the David software (david.abcc.ncifcrf.gov/) and further refined using the Genecard database (http://www.genecards.org) to prioritize genes for which a targeted agent could be available. The only exceptions were DICER1 and DROSHA, which were included to test the hypothesis that NACT changes the expression of the miRNA processing enzymes. Correlation between micro-RNAs and target genes was assessed using the Spearman correlation test, setting the threshold of significance for a p-value <0.05. If multiple microRNAs were present with significant capability of modulating gene expression, the microRNA with the lowest p-value was selected for the correlation analysis with gene expression.

For quantification in high vs. low expression of HGF/c-Met protein the cutoff was represented by the median value. All statistical analyses were performed with the JMP9 software package (SAS Institute).

## SUPPLEMENTARY METHODS, FIGURES AND TABLES


