# Supplementary material for: HGF/c-Met axis drives cancer aggressiveness in the neo-adjuvant setting of ovarian cancer
Source: Oncotarget. 2014 Jun 1;5(13):4855–67. doi: 10.18632/oncotarget.2049 (PMC4148105; doi:10.18632/oncotarget.2049)
Supplement: Supplementary file 1 [file oncotarget-05-4855-s001.pdf]

HGF/c-Met axis drives cancer aggressiveness in the neo-adjuvant setting of ovarian cancer

Supplementary Methods: Detailed Quantitative Fluorescent Immunohistochemistry protocol

Tissue Microarray (TMA) Construction: Tissue specimens were prepared in a TMA format: representative tumor areas were obtained from FFPE specimens of the primary tumor, and up to three representative replicate 2-mm cores from multiple tumor blocks were taken after review and marking of the hematoxylin and eosin stained slides by board-certified pathologists (SS. and PF). In total, 660 cores were taken and distributed over 11 slides from 109 patients, all of whom having pre and post-NACT paired samples. FFPE tissues used as controls included normal kidney, liver, brain, breast, lymph nodes, thymus, skeletal muscle and bladder along with breast cancer and non-small cell lung cancer.

Immunofluorescence: Quantitative fluorescent immunohistochemistry was performed for protein analysis. TMA slides were deparaffinized in xylene and then rehydrated in sequentially diluted ethanol solutions. Antigen retrieval was conducted by heating the slides in a steamer for 30 minutes in a solution of Tris-EDTA pH8.0. Endogenous peroxidase activity was blocked by treating the slides in Peroxidazed® reagent (Biocare Medical, Concord, CA) for 5 minutes. Non-specific binding was reduced by incubation with Background Sniper® (Biocare Medical, Concord, CA) for 10 minutes. Slides were incubated with the primary target antibodies and cytokeratin mask antibodies for HGF or c-Met diluted in Da Vinci Green antibody diluent (Biocare Medical, Concord, CA) for 1 hour at room temperature. Cyanine 5 (Cy5) directly conjugated to tyramide (Perkin-Elmer, Boston, MA) at a 1:50 dilution was used as the fluorescent detection for both target antigens.

HGF staining: A commercially available monoclonal anti-HGF antibody (clone H-10, Santa Cruz Biotech, Santa Cruz, CA) was diluted 1:100 in the Da Vinci Green solution which also included a rabbit anti-cow wide spectrum cytokeratin diluted 1:100. Specimens were incubated for 30 minutes with an Alexa Fluor® 555-conjugated anti-rabbit IgG to detect the anti-cytokeratin antibody, diluted 1:100 in the secondary Envision®+System-HRP polymer anti-mouse antibody (DAKO Developing System; DAKO, Carpinteria, CA). Cyanine 5 (Cy5) directly conjugated to tyramide was used for the specific fluorescent detection of HGF. HGF stained slides were then sequentially stained for CD68 (Santa Cruz Biotech, Santa Cruz, CA). CD68 mouse monoclonal antibody was incubated for 1 hour in TBS-T containing 0.1% BSA. Anti-CD68 was detected using Alexa Fluor® 488 conjugated goat anti-mouse IgG.

c-Met staining: A commercially available rabbit monoclonal anti-c-Met antibody (clone SP44) (Spring Bioscience, Pleasanton, CA) diluted 1:100 was used, with an anti-cytokeratin cocktail AE1/AE3 diluted 1:100 (Dako Carpinteria, CA) and an anti-chicken vimentin polyclonal antibody diluted 1:200 (Millipore, Temecula, CA). Specimens were incubated for 30 minutes with Alexa Fluor® 555-conjugated anti-mouse IgG diluted to 1:100, combined with an Alexa Fluor® 488-conjugated goat anti-chicken IgY diluted 1:100 in the Envision®+System-HRP polymer anti-rabbit antibody (DAKO Developing System; DAKO, Carpinteria, CA). Cyanine 5 (Cy5) directly conjugated to tyramide was used for the specific fluorescent detection of c-Met.

Automated Quantitative Analysis: Automated quantitative analysis (AQUA®) allows exact measurement of protein concentration within compartments of interest. In brief, a series of high-resolution digital images were captured by the ScanScope FL system (Aperio, Vista, CA). For each histospot, images were obtained using the signal from nuclei (4,6-Diamidino-2-phenylindole), CD68 (HGF staining) or vimentin (c-Met staining). Tumor was distinguished

from stromal or macrophage elements by creating an epithelial tumor “mask” from the cytokeratin signal and a stromal or macrophages “mask” from the vimentin or CD68 signals, respectively.

The images were analyzed by the AQUA® analysis software (Genoptix, Carlsbad, CA) to determine target protein expression in the epithelial, stromal, and macrophage cell populations by calculating the sum of target pixel intensity divided by the compartment area and normalized for exposure time.

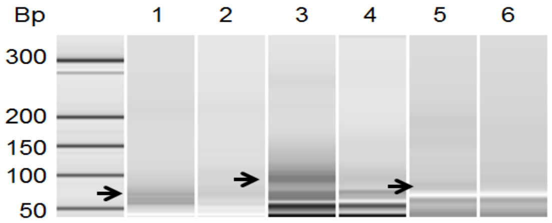

Supplementary Figure 1: Representative PCR analysis of HGF (lanes 1-2), MET (lanes 3-4) and TUBB (lanes 5-6) genes. Lanes 1, 3 and 5 were obtained from the library of cDNA pulled down with miR-193a-5p in OV2774 cells (10nm miR193a-5p, 48 hours of culture). Lanes 2, 4 and 6 were obtained from the negative control kept 48 hours with the transfecting medium. The expected PCR products were 72, 92 and 84, for HGF, MET and TUBB, respectively. Presence of the expected PCR products in lanes 1 and 3 are highlighted with the arrow, where no products are noticeable at the expected bp size in lanes 5 and 6.

Supplementary Tab. 1

Results of Wilcoxon test for the predicted targets of micro-RNA analyzed in the discovery setting.

Gene	P[1]	Fold Difference[2]	Inhibitor[3]	Micro-RNA[4]	Correlation[5]	P[6]
CCL2	0.0228	278.13%	Telmisartan	MIR-128a	0.3275	0.0073
HGF	0.005	249.73%	Rilotumumab	MIR-193a-5p	0.506	<.0001
CXCL12	0.0154	244.28%	Tinzaparin	MIR-27a	0.4283	0.0002
ID4	0.0337	224.49%	LY294002	Let-7g	0.2379	0.0474
CXCR4	0.0549	204.15%	AMD 3465	MIR-128a	0.5466	0.0001
PTEN	0.0203	193.23%	LY294002	Let-7c	0.3526	0.0017
GNAI1	0.006	191.64%	NF-023	MIR-193a-5p	0.3689	0.0099
MITF	0.0488	174.84%	Hinokitiol	MIR-141	-0.3375	0.0509
TGFB1	0.0082	61.90%	SB431542	MIR-145	-0.3033	0.0069
ERBB2	0.0385	57.89%	Lapatinib	MIR-221	-0.3743	0.0007
MET	0.0491	52.96%	Crizotinib	MIR-193a-5p	-0.338	0.0026
CHEK1	0.0016	41.60%	PF-477736	MIR-193a-5p	-0.296	0.0167
KIF11	0.0001	40.53%	Litronesib	MIR-141	0.4168	0.0142
TWIST1	0.0005	32.59%	QLT0267	MIR-193a-5p	0.2792	0.0267
PBK	0.0001	19.62%	ADP	MIR-145	-0.3113	0.0069
MKI67	0.0001	18.78%	Progesterone	MIR-145	-0.3618	0.0013
PLK1	0.0001	16.93%	Cyclapolin 9	MIR-193a-5p	-0.3688	0.0012
SRC	0.0175	2.81%	Dasatinib	Let-7c	-0.339	0.0066
DROSHA	0.0621	61.92%				
SEMA6D	0.0934	157.43%	Imatinib	MIR-320		
NEK6	0.1057	68.48%	PF-4708671	MIR-141		
CLOCK	0.1543	73.76%	Fluvoxamine	MIR-141		
ZEB1	0.1841	52.16%	Gefitinib	Let-7		
CYP39A1	0.1953	152.35%	Taurocholic acid	MIR-20		
HIPK1	0.1959	153.02%	Imatinib	MIR-143		
CXCL10	0.2124	22.80%	MDX-1100	Let-7		
FGFR1	0.2124	71.06%	AZD4547	MIR-125a		
CCND1	0.2233	66.08%	Estradiol	Let-7		
GLI3	0.2402	173.91%	XL139	MIR-141		
COL11A1	0.2442	47.60%	AEBSF	MIR-193a		
SOX2	0.2569	71.36%	Retinoic acid	MIR-320		
PDGFRB	0.2636	136.61%	Dasatinib	MIR-143		
EPHA3	0.2884	301.51%	PD98059	Let-7		
HOXB7	0.2927	57.89%	Retinoic acid	Let-7		
ID1	0.3099	77.85%	Retinoic acid	Let-7		
ADM	0.3355	63.73%	Omapatrilat	MIR-92		
CAMK2D	0.3564	236.98%	KN-93	MIR-145		
CTNNB1	0.3607	118.48%	SB415286	MIR-125a		
VAV3	0.367	71.82%	Abiraterone	MIR-145		
TUBB3	0.3906	147.78%	Ixabepilone	Let-7		

ZEB2	0.4138	149.30%	Gefitinib	MIR-193a
IGF1R	0.4241	79.70%	NVP-AEW541	MIR-320
INHBA	0.4242	66.54%	Sunitinib	Let-7
PIM1	0.4397	130.43%	SGI1776	MIR-320
MECP2	0.4424	83.50%	Azathioprine	MIR-141
ESR1	0.4588	77.22%	Fulvestrant	MIR-145
ROCK1	0.4651	114.94%	RKI-1447	MIR-145
HIF1A	0.4693	83.18%	NVPBEZ-235	MIR-125a
FES	0.4695	147.95%	TAE684	MIR-92
COL1A1	0.4705	148.52%	Imatininb	Let-7
BCL2	0.4912	118.62%	Navitoclax	MIR-125a
CCL22	0.4923	69.73%	SB203580	Let-7
TSC1	0.5014	74.86%	PD98059	MIR-27a
COX7A2L	0.5703	113.78%	Indanocine	MIR-125a
PPP2CA	0.6051	87.71%	Okadaic Acid	MIR-125a
MID1	0.6209	88.81%	Metformin	MIR-92
HIC2	0.6211	120.98%	DCA	MIR-27a
BMI1	0.6226	87.56%	Estradiol	MIR-27a
MAPRE1	0.6276	91.21%	Nocodazole	MIR-125a
PTK2	0.6279	93.36%	PF 573228	Let-7
DLC1	0.6366	129.80%	Ursodeoxycholic acid	MIR-141
CCL5	0.6506	84.69%	TAK-779	MIR-193a
AXL	0.6768	119.99%	BMS-777607	MIR-92
KLF7	0.681	111.54%	XL139	Let-7
BGN	0.6832	123.70%	Dexamethasone	MIR-193a
PPARGC1A	0.6857	125.05%	Troglitazone	MIR-221
ARNT2	0.712	91.11%	Fulvestrant	MIR-92
ESR2	0.7253	119.00%	PHTPP	MIR-125a
DICER1	0.7735	92.92%		
CHEK2	0.7977	92.45%	NSC 109555 ditosylate	MIR-320
EPAS1	0.8477	103.94%	PD98059	MIR-92
KDR	0.8517	105.11%	Sunitinib	MIR-221
JAK2	0.8666	104.06%	Ruxolitinib	MIR-221
FAP	0.8804	107.23%	Asunaprevir	MIR-141
GBP1	0.8889	94.43%	BMS345541	MIR-20
SHMT2	0.8926	97.56%	Mimosine	Let-7
AR	0.9191	97.09%	Bicalutamide	MIR-320
IGF2R	0.9351	98.17%	Retinoic acid	MIR-143
OSBPL3	0.9364	97.25%	Taurocholic acid	Let-7
GLI1	0.9701	102.49%	XL139	MIR-145
JAK1	0.9828	100.71%	Ruxolitinib	MIR-20
ATXN1	0.9927	99.72%	SB203580	MIR-125a

[1] P-value of the Wilcoxon test scoring the significance of the difference between PDS-CT vs. NACT group;

2 Fold difference calculated by averaging the results of the gene expression NACT vs. PDS-CT group. A

value higher and lower than 100% indicates increase of decrease, respectively, of the gene target in the NACT group; [3] Actionable inhibitor of the target gene identified through the Genecard and Pubmed database; [4] Micro-RNA with the highest correlation with the gene target; [5] R value of the Spearman gene :micro:RNA correlation; [6] P-value of the Spearman correlation presented in 5.
